# A comparison of emulsifiers for the formation of oil-in-water emulsions: stability of the emulsions within 9 h after production and MR signal properties

**DOI:** 10.1007/s10334-021-00970-9

**Published:** 2021-10-26

**Authors:** Victor Fritz, Petros Martirosian, Jürgen Machann, Rolf Daniels, Fritz Schick

**Affiliations:** 1 Section on Experimental Radiology, Department of Diagnostic and Interventional Radiology, University of Tuebingen, Tuebingen, Germany; 2 Institute for Diabetes Research and Metabolic Diseases of the Helmholtz Centre Munich at the University of Tuebingen, Tuebingen, Germany; 3 German Center for Diabetes Research (DZD), Neuherberg, Germany; 4 Institute of Pharmaceutical Technology, University of Tuebingen, Tuebingen, Germany

**Keywords:** Oil-in-water emulsions, Fat water MRI, Emulsifiers, Relaxometry, DWI

## Abstract

**Objective:**

To provide a basis for the selection of suitable emulsifiers in oil-in-water emulsions used as tissue analogs for MRI experiments. Three different emulsifiers were investigated with regard to their ability to stabilize tissue-like oil-in-water emulsions. Furthermore, MR signal properties of the emulsifiers themselves and influences on relaxation times and ADC values of the aqueous phase were investigated.

**Materials and methods:**

Polysorbate 60, sodium dodecyl sulfate (SDS) and soy lecithin were used as emulsifiers. MR characteristics of emulsifiers were assessed in aqueous solutions and their function as a stabilizer was examined in oil-in-water emulsions of varying fat content (10, 20, 30, 40, 50%). Stability and homogeneity of the oil-in-water emulsions were evaluated with a delay of 3 h and 9 h after preparation using T_1_ mapping and visual control. Signal properties of the emulsifiers were investigated by ^1^H-MRS in aqueous emulsifier solutions. Relaxometry and diffusion weighted MRI (DWI) were performed to investigate the effect of various emulsifier concentrations on relaxation times (T_1_ and T_2_) and ADC values of aqueous solutions.

**Results:**

Emulsions stabilized by polysorbate 60 or soy lecithin were stable and homogeneous across all tested fat fractions. In contrast, emulsions with SDS showed a significantly lower stability and homogeneity. Recorded T_1_ maps revealed marked creaming of oil droplets in almost all of the emulsions with SDS. The spectral analysis showed several additional signals for polysorbate and SDS. However, lecithin remained invisible in ^1^H-MRS. Relaxometry and DWI revealed different influences of the emulsifiers on water: Polysorbate and SDS showed only minor effects on relaxation times and ADC values of aqueous solutions, whereas lecithin showed a strong decrease in both relaxation times (r_1,lecithin_ = 0.11 wt.%^−1^ s^−1^, r_2,lecithin_ = 0.57 wt.%^−1^ s^−1^) and ADC value (Δ(ADC)_lecithin_ =  − 0.18 × 10^–3^ mm^2^/s⋅wt.%) with increasing concentration.

**Conclusion:**

Lecithin is suggested as the preferred emulsifier of oil-in-water emulsions in MRI as it shows a high stabilizing ability and remains invisible in MRI experiments. In addition, lecithin is suitable as an alternative means of adjusting relaxation times and ADC values of water.

## Introduction

Magnetic resonance imaging (MRI) provides a powerful non-invasive tool that enables the quantitative determination of various tissue properties, such as fat content, relaxation times and diffusivity. In recent years, huge efforts have been made to develop and optimize new imaging techniques with high contrast between different types of tissue. Furthermore, there is a clear tendency to use MRI for quantitative tissue characterization. The results of MRI examinations regarding relaxation properties, fat content of tissues (especially liver and pancreas, but also musculature) should be as consistent as possible in examinations on different MRI systems from different manufacturers. Only then are absolute values and results of follow-up examinations of patients on different MRI systems comparable. Reliable measurement phantoms are therefore indispensable for testing MRI equipment, and their composition and production have become an important area of research [1–4].

The main components of biological tissues are water and fat, which make emulsions particularly suitable for simulation of in vivo conditions. Emulsions are disperse systems consisting of at least two immiscible liquids, such as water and oil [5, 6]. They combine both, the properties of water and those of fat, and thus provide an excellent material for phantoms that simulate a variety of tissues in MRI, but also for studying changes in relaxation times and diffusivity of water and fat in the mixture.

**Fig. 1:**
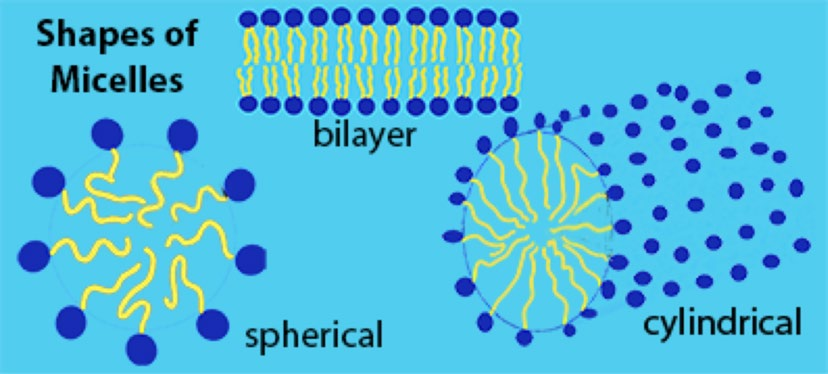
Schematic representation of different shapes of micelles in aqueous solutions (inspired by [7])

Unfortunately, emulsions are thermodynamically unstable by nature. Due to the hydrophobic nature of lipids and the resulting high interfacial tension, emulsions are short-lived and tend to separate in a pure water and a pure oil phase immediately after preparation. To produce a stable or at least long-lived emulsion, a third component, namely an emulsifier, has to be added. Emulsifiers are surface active agents (“surfactants”) that facilitate the formation of an emulsion and assist in their stabilization. This is due to their amphiphilic molecular structure. They consist of both, a hydrophilic head group and at least one non-hydrophilic hydrocarbon chain tail. Another fundamental characteristic of surfactants is the formation of aggregates, so-called micelles, in aqueous solution [7]. Micelles are complex structures that are formed due to the hydrophobic effect. Depending on the molecular structure of the emulsifier molecules, various shapes of micelles, such as spherical, cylindrical, bilayers, occur as depicted in Fig. 1.

A large number of surfactants (e.g., polysorbates, sodium dodecyl sulfate, soy lecithin) are utilized for industrial production of cosmetics, food, pharmaceuticals, and many other goods. Nevertheless, those substances are usually not present in human tissues and their MR signals might lead to undesired effects. Micellization of emulsifier molecules may influence the state of water and thus also MR parameters, such as relaxation times and diffusivity.

For a reliable simulation of tissue, it is essential that the emulsifiers used for stabilization have either none or precisely calculable effects on the MR measurements. Therefore, the influences that originate from the used emulsifiers should be well known prior to the measurements. Consequently, when selecting a suitable emulsifier, its MR signal properties and effects on MR properties of the solvent, e.g., relaxation times and diffusivity, should be taken into account.

Up till now, many MR scientists have already used oil-in-water (o/w) emulsions for validation and comparison of various water or fat MRI-based quantification methods [8–14], but also to study changes in the MR signal of water in the presence of fat and vice versa [15]. In MR technology, there are a number of different approaches for determining the fat content in tissue. In principle, a distinction is made between the very sensitive volume-selective ^1^H spectroscopy and imaging-based methods. Different sequences have been proposed and used as imaging techniques, mostly based on the different chemical shift of water and fat resonances. Currently, the so-called Dixon technique is mainly used to determine the fat content in tissue in clinical and experimental investigations. Different types of emulsifiers and thickeners have been used to stabilize the emulsions. However, to the best of our knowledge, the potential impact of surfactants on various MR parameters has not yet been systematically investigated.

The overall purpose of this work was to test common approaches and to derive a recommendation for the selection of a suitable emulsifier for use in MRI fat–water phantoms. For this, three different common emulsifiers were chosen and tested with regard to the following criteria: (1) Their ability to stabilize oil-in-water emulsions with the preparation method (ultrasound emulsification) presented in this study, (2) whether and which signal properties the emulsifiers show by themselves and (3) what influences they have on MR parameters, such as relaxation times and diffusivity of the aqueous phase. For these purposes, the emulsifiers were examined both as an additive in o/w emulsions and individually in aqueous solutions (without the presence of fat).

To assess the stabilizing ability of the emulsifiers, o/w emulsions of varying fat/water content (10/90, 20/80, 30/70, 40/60, 50/50%) were prepared and examined using T_1_ mapping. Due to the different relaxation properties of water and fat, T_1_ mapping provides a valuable tool for monitoring of phase separation or creaming occurring in emulsions. There are already some publications that have shown that MRI is a promising tool for assessing the stability and homogeneity of emulsions [16, 17].

For determination of signal properties of the emulsifiers, aqueous emulsifier solutions were investigated in proton-MR spectroscopy (^1^H-MRS) experiments. Effects of various emulsifier concentrations on relaxation times and apparent diffusion coefficient (ADC) values of aqueous emulsifier solutions were investigated by relaxometry (T_1_ and T_2_ mapping) and diffusion weighted MRI.

## Materials and methods

### Data acquisition and analysis

Imaging and spectroscopy were performed on a clinical-, whole-body 3.0 T MR system (MAGNETOM Prisma^fit^, Siemens Healthineers, Erlangen, Germany) using a 20-channel head coil. All data were collected at room temperature (22 °C) and processed offline with MATLAB software (MathWorks, Natick, MA).

### Choice of emulsifiers

Polysorbate 60 (Kolliphor^®^ PS 60, BASF, Ludwigshafen, Germany), sodium dodecyl sulfate (SDS, Carl Roth, Karlsruhe, Germany) and soy lecithin (Carl Roth, Karlsruhe, Germany) were used as emulsifiers and trialed individually. The cost of each emulsifier is around 20–30 euros per 250 g.

Polysorbate 60 is a non-ionic surfactant and emulsifier derived from polyethoxylated sorbitan and stearic acid (polyoxyethylene (20) sorbitan monostearate). Polysorbates are widely used as a solubilizer and emulsifier in pharmaceuticals and food. It shows excellent emulsifying properties and low toxicity.

Sodium dodecyl sulfate (SDS) is an anionic surfactant, consisting of a hydrocarbon chain with a sulfate anion at its end and a sodium cation (C_12_H_25_NaO_4_S). SDS is mainly used in detergents and cosmetics.

Lecithins are phospholipids and form the group of emulsifiers most commonly used in the food industry. Lecithins are naturally occurring emulsifiers and the building block of biological membranes in animal and plant cells. They consist of fatty acids (two hydrocarbon chains), phosphoric acid, glycerol and choline.

### Preparation of oil-in-water emulsions

For testing the emulsifiers, oil-in-water emulsions of varying fat/water content (10/90, 20/80, 30/70, 40/60, 50/50%) were prepared in volumes of 50 ml. Emulsions without any additives were prepared additionally. Peanut oil was used as dispersed phase because of its nuclear magnetic resonance spectrum which is similar to that of triglycerides in human adipose tissues [18]. The aqueous phase was first prepared by completely dissolving the emulsifiers in distilled water. The weight fraction of the emulsifier was fixed at 10% with respect to the oil phase for each emulsion (m_emulsifier_/m_oil_ = 0.1). To increase solubility, the aqueous phase was heated up to 30–40 °C using a water bath.

**Fig. 2:**
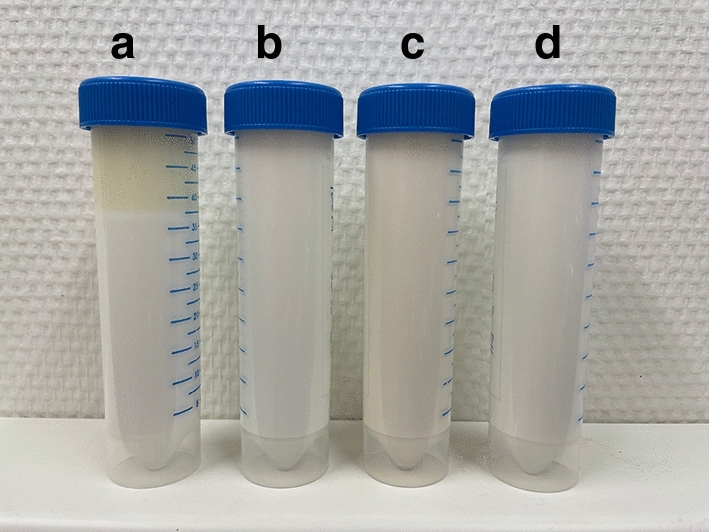
Photograph of emulsions with a fat content of 30% immediately after their preparation. Different emulsifiers were used for stabilization (**a**: no emulsifier, **b**: polysorbate 60, **c**: soy lecithin, **d**: SDS)

Coarse emulsions (“premixes”) were prepared by adding the peanut oil into the aqueous phase under gentle stirring. These premixes were emulsified by ultra-sonication using UP200Ht with S26d14 sonotrode (Hielscher Ultrasonics, Teltow, Germany). Ultrasonic emulsification is very common, as it offers short preparation times, easy handling and resulting stable and fine emulsions [19]. The sonication time was 90–120 s with an output of 70 W. First, the premixes for each emulsifier and each fat fraction were prepared and then all samples were emulsified by ultra-sonication. The total preparation time for all 20 emulsions was approx. 4 h. All emulsions were stored in CELLSTAR polypropylene tubes (Greiner Bio-One, Frickenhausen, Germany) at room temperature until analysis. Figure 2 shows a photograph of the emulsions with a fat content of 30%.

### Stability analysis of emulsions

MR examinations were carried out approximately 3 h and 9 h after preparation of the emulsions. Since emulsions are hardly completely stable for several weeks or months, it was important for us to test the stability of the emulsions in the practical range of a few hours after production. The components water and oil show pronounced T_1_ differences and thus the decomposition of the emulsion leads to a dispersion of measured T_1_ values obtained by mono-exponential fitting along the gravity axis. Thus, spatially resolved T_1_ mapping was applied for estimation of the temporal–spatial stability of the emulsions. The T_1_ maps were calculated based on signal amplitudes from inversion recovery measurements by an IR-TSE sequence with seven different TIs ranging from 25 to 8000 ms. TR and TE were set to 10,000 ms and 9.9 ms, respectively. A three-parameter model was used for pixelwise mono-exponential fitting of the measured signal intensities. It should be mentioned that quantitative T_1_ values obtained by mono-exponential fitting are neither correct for water nor for fat, but for the fat/water mixture. Resulting T_1_ values were only used as sensitive markers to assess the stability of the emulsions. In addition to the MR examinations, all emulsions were visually controlled over a storage time of a maximum of 48 h.

### Spectroscopic characterization of the emulsifiers

To determine whether the emulsifiers themselves lead to detectable MR signals, an aqueous emulsifier solution (3 wt.%) of each emulsifier was spectrally analyzed. MR spectra were recorded using a Stimulated Echo Acquisition Mode (STEAM) sequence with TR of 10,000 ms and TE of 10 ms. For each spectrum, 8 acquisitions were taken from a 10 × 10 × 10 mm cubic volume in the center of the sample. Reference spectra of distilled water and peanut oil were recorded to identify possible emulsifier signals in emulsions. Post-processing of spectra, such as apodization, and baseline and phase correction, were performed using jMRUI software (http://www.jmrui.eu) [20, 21].

### Influence of the emulsifier concentration on the relaxation times of the aqueous phase

**Fig. 3:**
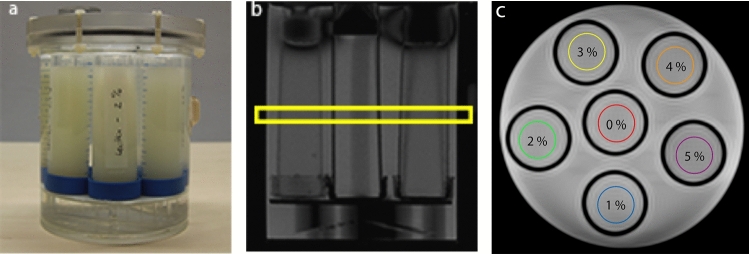
(**a**) Photograph of the cylindrical MR phantom with CELLSTAR tubes containing emulsifier solutions of varying concentrations. The surroundings of the tubes in the phantom were filled with water to reduce susceptibility effects at the walls of the tubes. (**b**) Localizer image with selected slice for T_1_ and T_2_ mapping. (**c**) Localizer image to illustrate the arrangement of the aqueous emulsifier solutions in the cylindrical MR phantom

Relaxometric measurements were performed to investigate whether and to what extent the presence of emulsifiers might modify the relaxation times of the aqueous phase. For this purpose, T_1_ and T_2_ mapping sequences were applied on aqueous emulsifier solutions of concentrations with 0, 1, 2, 3, 4, and 5 wt.% (without the presence of fat). The solutions were stored in CELLSTAR tubes and fixed in a cylindrical phantom as shown in Fig. 3a.

Quantitative T_1_ mapping was performed using the same scanning protocol as for the stability analysis described above. Quantitative T_2_ maps were acquired using a Carr-Purcell-Meiboom-Gill (CPMG) spin-echo pulse sequence with non-selective refocusing pulses. TR was 6000 ms and images with 32 different TEs ranging from 50 to 1600 ms (increment: 50 ms) were recorded. For T_1_ and T_2_ measurements, the selected slice orientation was coronal as shown in Fig. 3b, c.

T_1_ and T_2_ maps were calculated pixel by pixel and representative relaxation times for the solutions were determined from regions of interest (ROIs) within each sample. Following this, measured relaxation times (T_1_, T_2_) and relaxation rates (*R*_1_ = 1/T_1_, *R*_2_ = 1/T_2_) were plotted as a function of emulsifier concentration. From the slope of the straight line functions in these plots, relaxivities *r*_1_ = ΔR_1_ and *r*_2_ = ΔR_2_ were derived for each emulsifier.

### Influence of the emulsifier concentration on the diffusivity of the aqueous phase

Emulsifier effects on diffusivity of water were measured as a function of the emulsifier concentration. The same samples were used as for the relaxation time measurements.

Diffusion-weighted imaging (DWI) was performed using a readout-segmented echo planar imaging sequence in 3-scan-trace mode with monopolar diffusion gradients, four different b values (0, 50, 500, 1000), and coronal slice orientation. TR and TE were set to 5000 ms and 51 ms, respectively. For polysorbate and SDS, DWI data were acquired with an additional inversion recovery magnetization preparation (TI_poly_ = 240 ms, TI_SDS_ = 300 ms) to avoid interfering signal components caused by the emulsifiers. ADC maps were calculated from the acquisitions with multiple *b* values using a log-linear fitting of the signal intensities. In analogy to the relaxation time measurements, ADC values were determined in a ROI within each sample and plotted as a function of the emulsifier concentration.

## Results

### Stability analysis

**Fig. 4:**
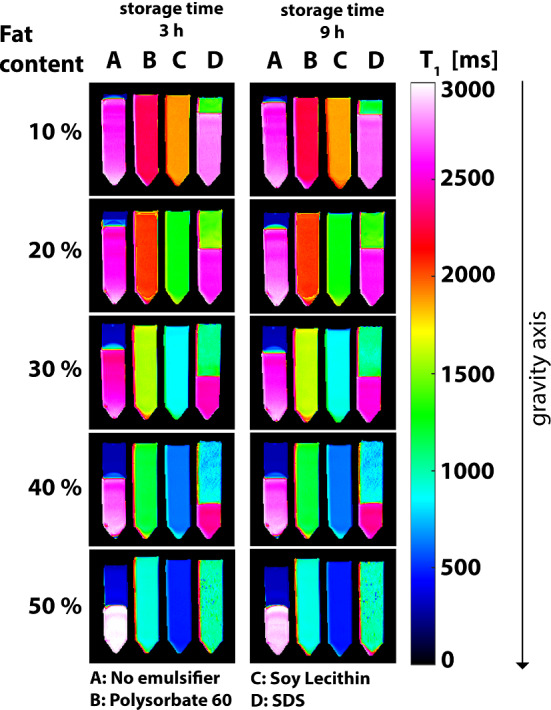
T_1_ maps of oil-in-water emulsions with different emulsifiers (**A**: no emulsifier, **B**: polysorbate 60, **C**: soy lecithin, **D**: SDS) and with increasing fat content (from 10% to 50%) from top to bottom. Measurements were carried out after storage times of approx. 3 h and 9 h, respectively. The presented T_1_ values were obtained by mono-exponential fitting and correspond to the T_1_ values of the oil/water mixture

T_1_ mapping provided high sensitivity to segregation processes in the samples. Figure 4 displays the T_1_ maps of the oil-in-water emulsions for the three emulsifiers, regarding samples with increasing fat content from top to bottom. Hardly any differences were found between a storage time of 3 h and a storage time of 9 h.

As expected, emulsions without any additives (column: A) did not mix and tended to separate into a pure water (higher T_1_) and pure oil phase (lower T_1_) immediately after preparation. Results could be used as a benchmark for a stability assessment of emulsions prepared with polysorbate 60, SDS and lecithin.

Emulsions prepared with Polysorbate 60 (column: B) and soy lecithin (column: C) show a spatially very homogeneous T_1_ distributions over the entire samples, regardless of fat content and storage time. Thus, all emulsions stabilized by polysorbate or lecithin were stable and homogeneous, and none of those showed marked decomposition or creaming within the first 9 h. An interesting finding was a distinct difference between T_1_ values of emulsions with the same composition of water and oil, but different emulsifiers: Emulsions with Polysorbate 60 (column: B) showed clearly longer T_1_ values than those with soy lecithin (column: C).

In contrast to the successful stabilization of emulsions in columns B and C, emulsions using SDS as emulsifier (column: D) showed a significantly lower stability and homogeneity. With the exception of the sample with a fat content of 50%, emulsions with SDS had a separation into two distinct phases, indicated by areas with different T_1_ in the map. The phase separation was caused by the gravitational creaming of oil droplets. This means that the oil droplets individually rose to the top of the containers (so-called “creaming”), which obviously results in two distinct phases, an upper oil-rich phase (lower T_1_) and a lower water-rich phase (higher T_1_) within the emulsions. However, a complete separation or a break into a pure water and pure oil phase, as in the case of the emulsions in column A, was not observed in any of the emulsions with SDS. Emulsions with a fat content of 50% were the most stable and homogeneous for SDS. No creaming was observed. But even those does not appear (by means of visual control) as homogeneous compared to those emulsions prepared with polysorbate or lecithin.

After a storage time of 48 h, at least a slight creaming was visible to the eye in almost all of the emulsions.

### Spectroscopic characterization of the emulsifiers

**Fig. 5:**
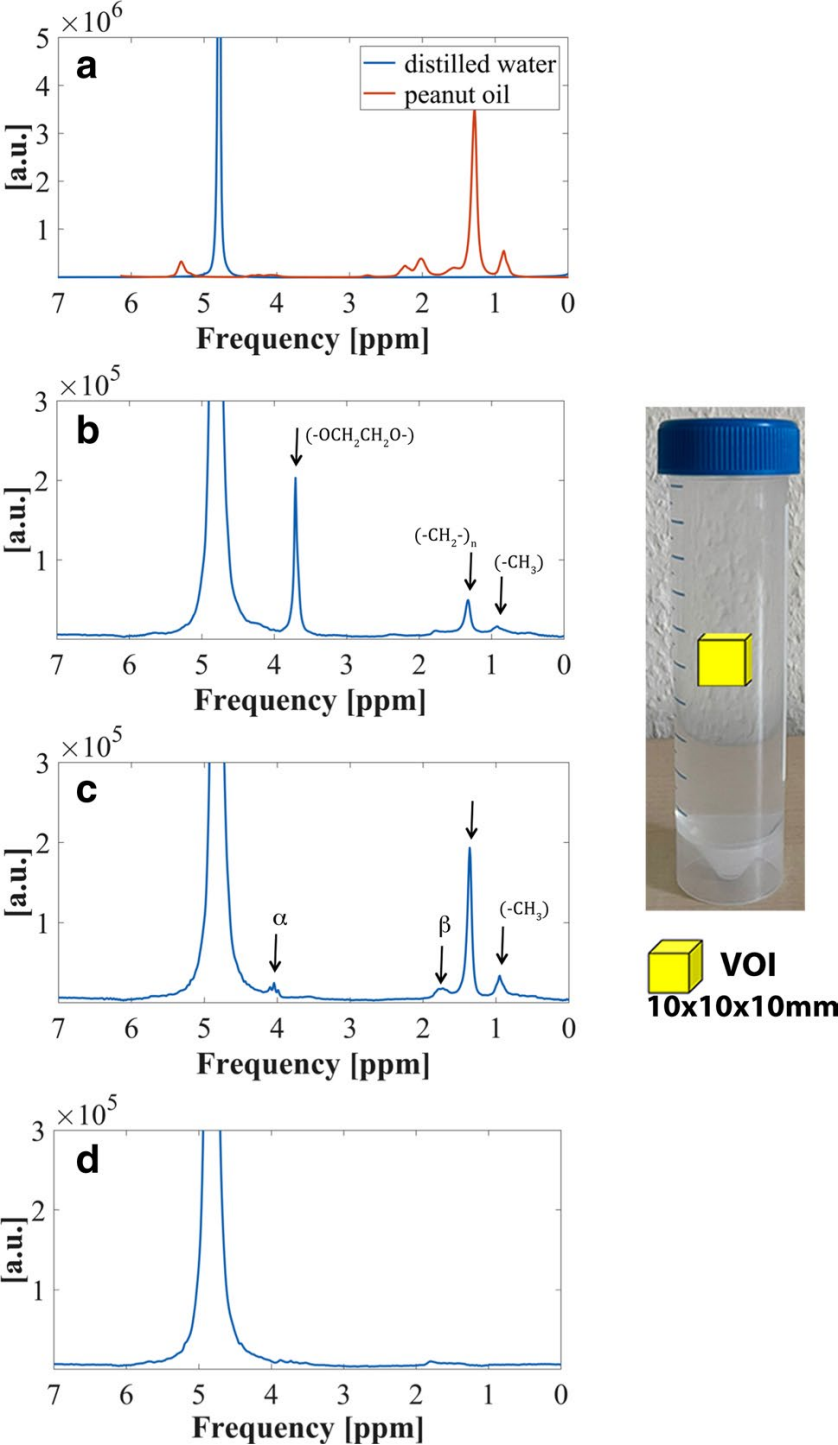
^1^H-MR spectra of (**a**) peanut oil in red color and distilled water in blue color (**b**) aqueous solution of polysorbate (3 wt.%) (**c**) aqueous solution of SDS (3 wt.%) (d) aqueous solution of lecithin (3 wt.%). Arrows indicate resonances of emulsifiers. The Voxel of interest (VOI) was positioned in the center of the samples

The results of the spectroscopic examinations of aqueous solution of the emulsifiers are presented in Fig. 5. Several signals (besides those of water and lipids) could be observed for polysorbate and SDS (indicated by arrows in Fig. 5b, c). Both show resonances at 1.3 ppm and 0.9 ppm. These signals can be assigned to methylene- (-CH_2_-)_n_ and terminal methyl protons (-CH_3_) and coincide with the signals of triglycerides in peanut oil (Fig. 5a).

Polysorbate leads to another dominant signal contribution at 3.7 ppm (Fig. 5b). For SDS, two further resonances of relatively low amplitudes were detected at 1.7 ppm and 4.2 ppm (Fig. 5c). For lecithin, no additional signals could be observed (Fig. 5d).

### Influence of the emulsifier concentration on the relaxation times of the aqueous phase

**Fig. 6:**
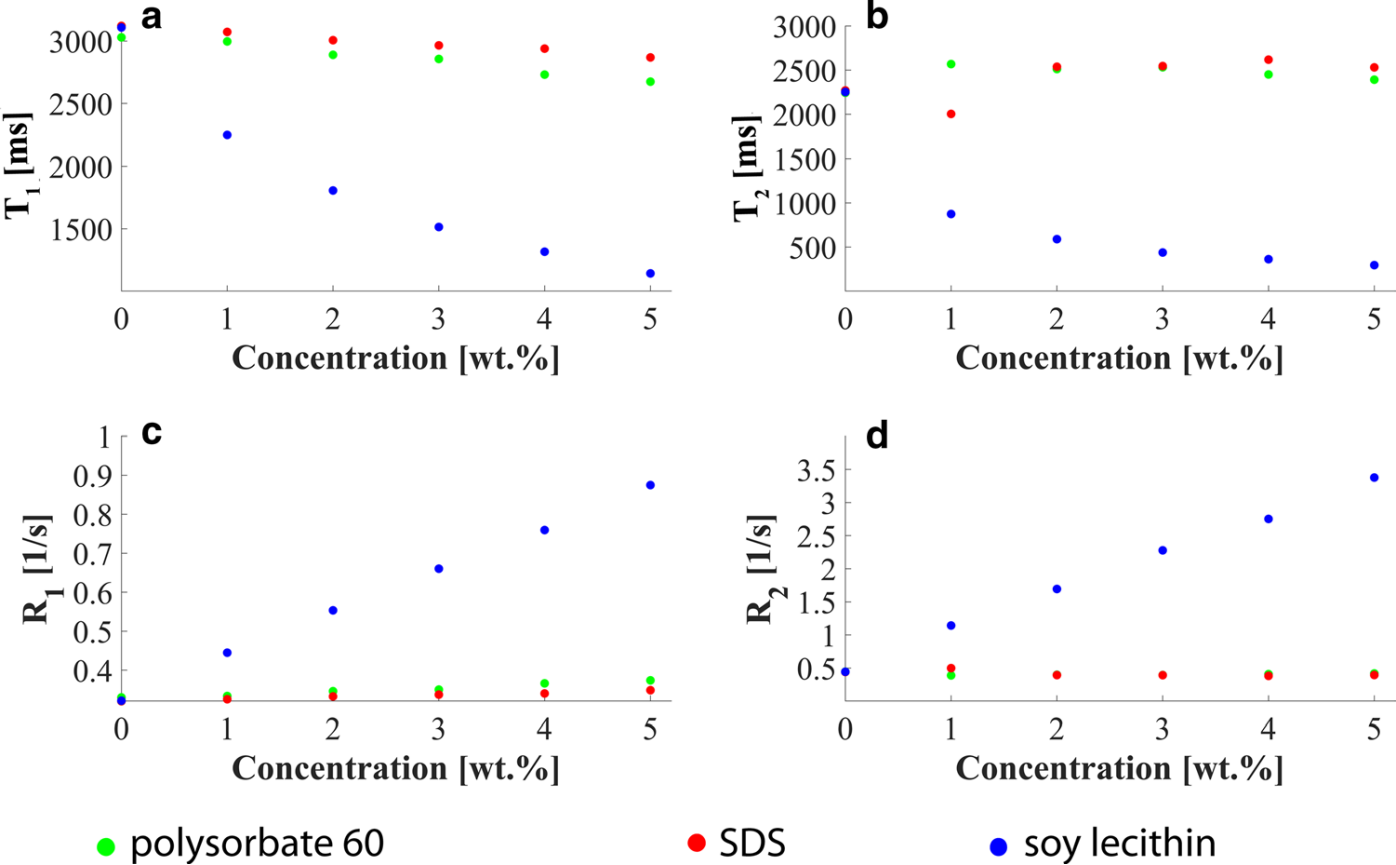
Dependence of relaxation properties on concentration of emulsifiers in distilled water: (**a**) T_1_ relaxation times, (**b**) T_2_ relaxation times (**c**) R_1_ relaxation rates and (**d**) R_2_ relaxation rates of aqueous solutions as a function of emulsifier concentration for polysorbate (green), SDS (red) and lecithin (blue)

Measured relaxation times (T_1_, T_2_) and the relaxation rates (R_1_, R_2_) of aqueous solutions are presented in Fig. 6a-d as a function of the concentration for all three emulsifiers investigated. Depending on the type of emulsifier, different influences on the relaxation times and rates were identified.

Lecithin (blue data points) showed a strong decrease in T_1_ and T_2_ with increasing concentration. There was a linear relationship between the relaxation rates (R_1_, R_2_) and the concentration of dissolved lecithin. The longitudinal and transversal relaxivities were determined to be r_1lecithin_ = 0.11 wt.%^−1^ s^−1^ (*R*^2^ = 0.99) and r_2lecithin_ = 0.57 wt.%^−1^ s^−1^ (*R*^2^ = 0.99).

In contrast, polysorbate and SDS (green and red data points) only resulted in slight shortening of T_1_ with increasing concentration. The longitudinal relaxivities were r_1polysorbate_ = 0.01 wt.%^−1^ s^−1^ (*R*^2^ = 0.97) for polysorbate and r_1SDS_ = 0.005 wt.%^−1^ s^−1^ (*R*^2^ = 0.98) for SDS. The transverse relaxation time did not show any clear dependence on the emulsifier concentration at all.

### Influence of the emulsifier concentration on the diffusivity of the aqueous phase

**Fig. 7:**
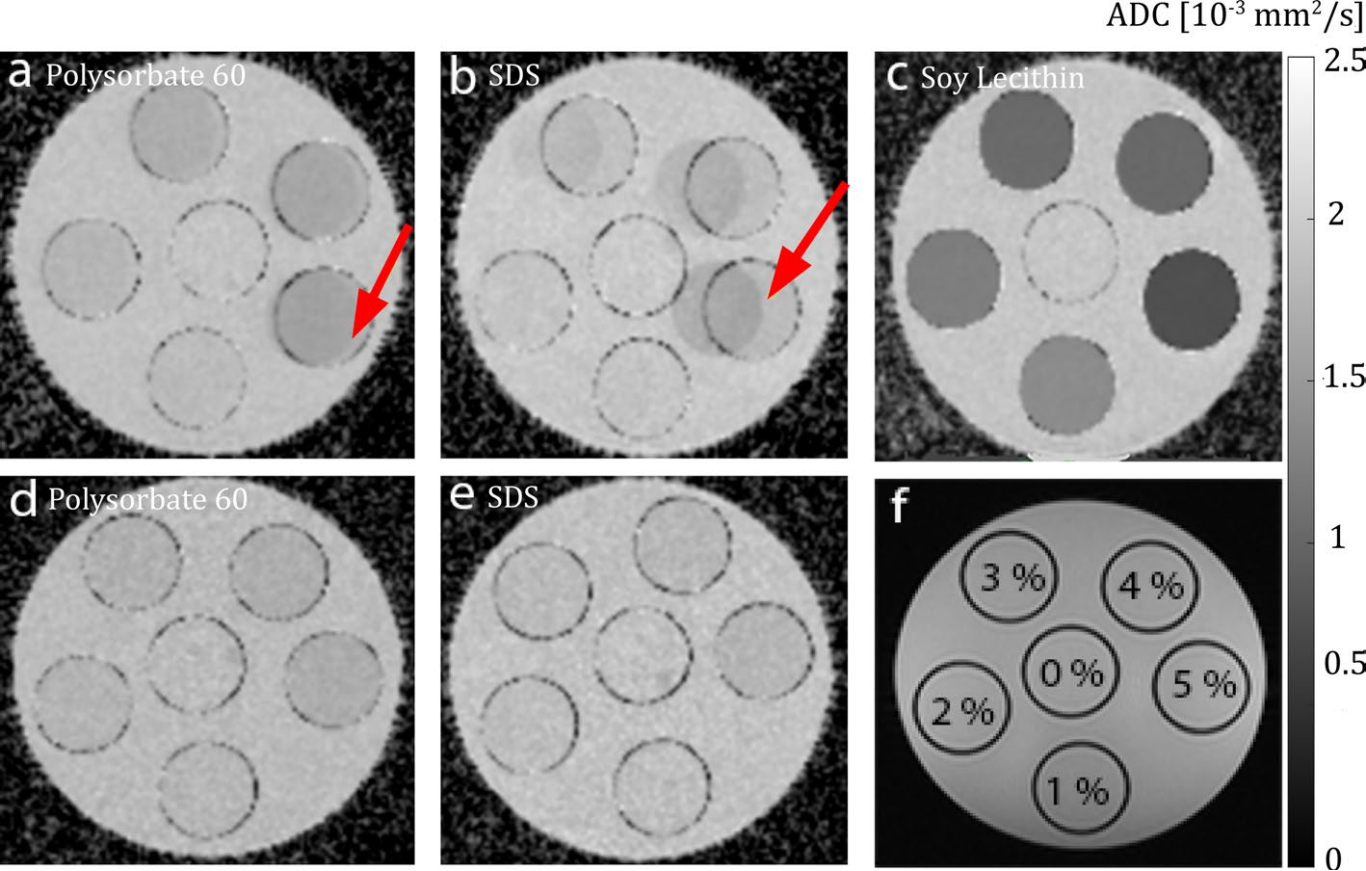
ADC maps of aqueous emulsifier solutions. The solutions were stored in CELLSTAR tubes and fixed in a cylindrical MR phantom. A central slice through the phantom is shown. (**a**–**c**) ADC maps obtained without additional inversion recovery preparation to suppress signal components caused by the emulsifiers (**a**: polysorbate, **b**: SDS, c: lecithin). Red arrows indicate chemical shift artifacts caused by the emulsifiers. (**d**–**e**) ADC maps acquired using an additional inversion recovery preparation for suppression of signal contributions from the emulsifiers themselves (**d**: polysorbate, **e**: SDS). (**f**) The arrangement of the samples with different emulsifier concentration in the MR phantom is depicted

ADC maps of aqueous emulsifier solutions are shown in Fig. 7, with the arrangement of the sample tubes regarding their concentration depicted in Fig. 7f. ADC maps derived from original images recorded without an additional inversion pulse show chemical shift artifacts in solutions with polysorbate and SDS (arrows in Figs. 7a, b). These effects originate from relatively strong signals of these emulsifiers at 3.7 ppm in polysorbate and 1.3 ppm in SDS (see spectral analysis). No artifacts were present when using an adapted inversion recovery preparation for nulling the frequency shifted signal contributions (Figs. 7d, e).

**Fig. 8:**
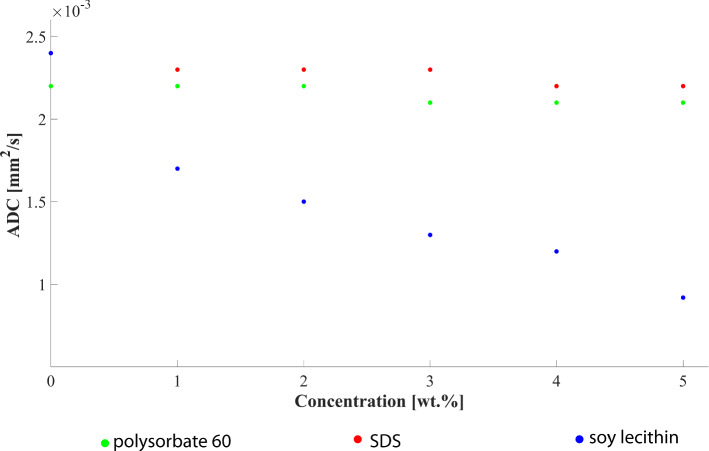
ADC values of water as a function of emulsifier concentration for polysorbate (green), SDS (red) and lecithin (blue)

For all aqueous solutions with emulsifiers, the ADC value of water decreased nearly linearly with increasing emulsifier concentration (Fig. 8), with the ADC reduction being most pronounced for lecithin [Δ(ADC)_lecithin_ =  − 0.18 × 10^–3^ mm^2^/s⋅wt.% (*R*^2^ = 0.95)]. Significantly weaker effects on ADC values were found for polysorbate and SDS with Δ(ADC)_polysorbate_ =  − 0.035 × 10^–3^ mm^2^/s⋅wt.% (*R*^2^ = 0.94) and Δ(ADC)_SDS_ =  − 0.02 × 10^–3^ mm^2^/s⋅wt.% (*R*^2^ = 0.99), respectively.

## Discussion and conclusion

The presented work checked the suitability of three different emulsifiers for the production of emulsions for tissue-like and/or sequence calibration phantoms. The stabilizing ability of the emulsifiers was evaluated in emulsions with an oil content up to of 50%. Furthermore, influences of the emulsifiers on the spectrum of detectable MR signals and on relaxation and diffusion properties of water in which the emulsifiers are dissolved were investigated. Reproducibility was not tested systematically, but most phantom procedures were carried out several times with very similar results.

Stability of emulsions could be achieved using polysorbate 60 or soy lecithin as stabilizers. Both emulsifiers showed a high stabilizing ability with the ultrasound preparation method. No creaming was observed within the first 9 h. Emulsions with SDS were found to be the ones with the lowest stability in this study. Already after a storage time of 3 h, recorded T_1_ maps (and visual inspection) revealed marked creaming of oil droplets in almost all of the emulsions with SDS. Since the rate of creaming is directly proportional to the square of the droplet size, it can be assumed that the droplet size achieved in the preparation process was significantly larger than that in emulsions stabilized by polysorbate or lecithin. Exact creaming rates can be determined by further measurements at earlier points in time, which was not done in our study. However, decomposition in our preparation scheme was much too fast for SDS. A reason for rapid decomposition seems to be the preparation process itself, since other research groups, for example, Bernard et al. [8], reported very stable fat–water phantoms using SDS. However, they added carrageenan as a gelling agent, which additionally increases the stability of the samples. Another reason could be the selection of the lipid phase. The mentioned group used soya oil instead of peanut oil, which has a different fatty acid composition and could lead to a modified effect of the emulsifier. For these reasons, SDS was included in the rest of the study.

Spectroscopic measurements of aqueous solutions with emulsifiers showed additional signals for polysorbate at 0.9 ppm, 1.3 ppm, and 3.7 ppm and for SDS at 0.9 ppm, 1.3 ppm, 1.7 ppm, and 4.2 ppm. Similar results were reported in previous spectrometer studies [22, 23]. Especially the resonances at 1.3 ppm and 0.9 ppm, which are usually seen in triglycerides, are considered to be critical with regard to fat quantification. Other resonances located at 3.7 ppm for polysorbate and at 1.7 ppm and 4.2 ppm for SDS do not coincide with relevant fat signals, but influences of these signals on quantitative MR imaging cannot be ruled out and require further investigations. Even though the molecular structure of lecithin suggests signals in the ^1^H-MR spectrum, lecithin remained invisible in our spectroscopic examinations with TE = 10 ms. Lecithins are phospholipids consisting of a hydrophilic head group and two hydrophobic hydrocarbon chains (C_m_H_n_). One possible explanation for MR invisibility is the micellar structure of lecithin: In aqueous solution, lecithin forms so-called bilayers (shown in Fig. 1), similar to those of phospholipids in the cell membranes of animal cells. Due to the restricted mobility within such bilayer structures, the lipids have a very short relaxation time (T_2_ ~ 10^–2^ ms) and are therefore invisible for ^1^H-MRS measurements with TE values in the range of a few milliseconds [24, 25].

Relaxometric measurements of the aqueous solutions revealed partly considerable effects of emulsifiers on relaxation properties of water. However, for polysorbate and SDS, influences on relaxation times T_1_ and especially T_2_ were only minor. In both cases, the addition of the emulsifier led to a slight decrease in T_1_ for higher concentrations (r_1polysorbate_ = 0.01 wt.%^−1^ s^−1^, r_1SDS_ = 0.005 wt.%^−1^ s^−1^). A meaningful evaluation for transverse relaxivity r_2_ was not possible for polysorbate and SDS, as neither a steady decrease nor a steady increase in T_2_ was observed along the concentration axis. The samples with lecithin showed a completely different behavior: a comparatively strong decline in T_1_ and T_2_ was observed with increasing concentration, with relaxivities of r_1lecithin_ = 0.11 wt.%^−1^ s^−1^ and r_2lecithin_ = 0.57 wt.%^−1^ s^−1^. This interesting finding implies that lecithin might also provide an alternative agent for modification of relaxation times. Commonly, paramagnetic additives as Gd compounds or nickel or manganese ions, but also to some extend polysaccharides as agar or agarose are utilized as T_2_ and T_1_ modifiers [3, 26–28]. Unfortunately, these substances are partially toxic or require high temperature to be solved.

DWI experiments investigated potential restrictions of the mobility of water molecules by emulsifiers in aqueous solution. For all three emulsifiers, the ADC value of water in the solutions decreased with increasing concentration of the emulsifier. The probable cause for the decrease is the restriction of the water diffusivity by aggregates of emulsifier molecules. In aqueous solution, emulsifier molecules form their own organizational forms, the micelles, which represent barriers for the water molecules and restrict their mobility. With an increasing concentration of emulsifier molecules, the number of micelles and associated impairment of diffusivity increases. The greatest effect per amount of emulsifier was observed for lecithin with a decrease in the ADC value of Δ(ADC)_lecithin_ =  − 0.18 ⋅ 10^–3^ mm^2^/s per percent emulsifier by weight. This distinct decrease in the measured ADC values and the fact that lecithin does not show any detectable signals in ^1^H spectra or artifacts in MR images makes this substance particularly interesting for the construction of novel DWI phantoms. Polysorbate and SDS, on the other hand, showed significantly less influence on water diffusion properties at the same concentrations. For the latter, chemical shift artifacts in the diffusion images and ADC maps proved to be disruptive. Obvious chemical shift artifacts can be traced back to the signal components of the emulsifiers and are therefore consistent with the results from the spectral analysis.

In conclusion, lecithin is suggested as the preferred emulsifier for use in MRI, especially when common ultrasound emulsification is applied. Lecithin is non-hazardous, inexpensive and easy to handle. Use of lecithin as emulsifier not only provides a high stabilizing ability but also remains invisible in MRI experiments. The latter is of great importance when simulating MR properties of tissues by emulsions: additional signals are undesired and cause inaccuracies or even systematic errors in quantitative measurements. Besides its function as a stabilizer for o/w emulsions in fat–water phantoms, lecithin may provide an attractive agent for modifying T_1_ and T_2_ values or for adjusting diffusion properties. These features seem particularly interesting for matching relaxation times and/or ADC values to those of human tissues. 
